# 2187. Earliest Description of Mobile Genetic Elements (MGEs) Involved in Establishing *bla*_NDM_ Persistence Following its Spread From *Acinetobacter* spp. to Enterobacterales

**DOI:** 10.1093/ofid/ofad500.1809

**Published:** 2023-11-27

**Authors:** Dalila Touati, Frédéric Grenier, Simon Lévesque, Sebastien Rodrigue, Louis-Patrick Haraoui

**Affiliations:** Université de Sherbrooke, Sherbrooke, Quebec, Canada; Université de Sherbrooke, Sherbrooke, Quebec, Canada; Université de Sherbrooke, Sherbrooke, Quebec, Canada; Université de Sherbrooke, Sherbrooke, Quebec, Canada; Université de Sherbrooke, Sherbrooke, Quebec, Canada

## Abstract

**Background:**

*bla*_NDM_ is the most prevalent carbapenemase worldwide. It originated in *Acinetobacter* spp. and spread to Enterobacterales on MGEs despite bottlenecks in MGE transfers between, and persistence within, these phylogenetically distinct bacteria. To elucidate how this transfer and successful expansion were achieved, we investigated MGEs among the earliest NDM-positive Gram-negative bacteria (GNB), isolated in India in 2007.

**Methods:**

Thirteen polymerase chain reaction-confirmed NDM-positive GNB underwent whole-genome sequencing (Illumina NovaSeq and Oxford Nanopore Technologies MinION) and computational analyses: assembly (Unicycler and Trycycler), annotation (RAST), identification (Kraken2), plasmid incompatibility (PlasmidFinder).

**Results:**

Single copies of *bla*_NDM-1_ were found in 12 Enterobacterales (6 *K. pneumoniae*; 3 *E. coli*; 2 *E. cloacae*; 1 *E. hormaechei*) and 1 *A. baumannii* (Ab), located on plasmids in 11/13 strains: 6 IncC, 3 IncFII, 2 IncFIB (Figure 1). Partial or complete IS*Aba125* was found upstream of all *bla*_NDM_, with a second complete downstream copy forming Tn*125* only in Ab. *bla*_NDM_'s genetic environment was highly diverse including the earliest evidence of its association with MGEs that participated in its global dissemination: insertion sequence common region 1 (ISCR1, n=5) and Tn*3000* (n=4). IS*3000*, present in 2 copies bracketing Tn*3000*, was also found in single copies in 4 strains at varying distances upstream of *bla*_NDM_. In 3/4 of these, ISCR1 was downstream of *bla*_NDM_. These hybrid constructs may represent precursors leading to the formation of these MGEs, or degenerate structures.

Figure 1: Genetic environment surrounding blaNDM in 13 Gram-negative bacteria isolated in India in 2007.

**Figure:**
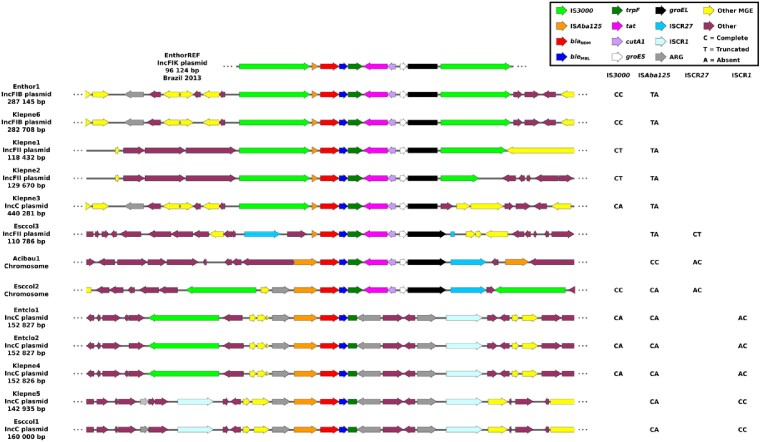

Acibau: Acinetobacter baumannii; Entclo: Enterobacter cloacae; Enthor: Enterobacter hormaechei; Esccol: Escherichia coli; Klepne: Klebsiella pneumoniae.

**Conclusion:**

We present evidence of early, rapid and diverse adaptation of *bla*_NDM_’s genetic environment following its transfer from *Acinetobacter* spp. to Enterobacterales. We identify for the first time potentially intermediate genetic structures in the transition between the ancestral Tn*125* structure from *Acinetobacter* spp., and both ISCR1 and Tn*3000,* which overtook Tn*125* as the predominant MGEs among NDM-positive Enterobacterales. Additional sequencing of early NDM-positive isolates could shed more light on the genetic events leading to the formation of MGEs associated with *bla*_NDM_, furthering our understanding of the emergence of MGEs carrying antibiotic-resistance genes.

**Disclosures:**

**All Authors**: No reported disclosures

